# Rapid monitoring of flavonoid content in sweet tea (*Lithocarpus litseifolius* (Hance) Chun) leaves using NIR spectroscopy

**DOI:** 10.1186/s13007-022-00878-y

**Published:** 2022-04-02

**Authors:** Zhaoxia Tian, Zifeng Tan, Yanjie Li, Zhiling Yang

**Affiliations:** 1 Research Institute of Subtropical Forestry, Chinese Academy of Forestry, No. 73, Daqiao Road, Fuyang, Hangzhou, 311400 Zhejiang Province China; 2 College of Forestry, Nanjing Forestry University, Nanjing, People’s Republic of China

**Keywords:** Sweet tea, Flavonoids content, Near-infrared (NIR) spectroscopy, Partial least squares (PLS) model, Model calibration

## Abstract

**Background:**

Sweet tea, which functions as tea, sugar and medicine, was listed as a new food resource in 2017. Flavonoids are the main medicinal components in sweet tea and have significant pharmacological activities. Therefore, the quality of sweet tea is related to the content of flavonoids. Flavonoid content in plants is normally determined by time-consuming and expensive chemical analyses. The aim of this study was to develop a methodology to measure three constituents of flavonoids, namely, total flavonoids, phloridin and trilobatin, in sweet tea leaves using near-infrared spectroscopy (NIR).

**Results:**

In this study, we demonstrated that the combination of principal component analysis (PCA) and NIR spectroscopy can distinguish sweet tea from different locations. In addition, different spectral preprocessing methods are used to establish partial least squares (PLS) models between spectral information and the content of the three constituents. The best total flavonoid prediction model was obtained with NIR spectra preprocessed with Savitzky–Golay combined with second derivatives (SG + D2) (R_P_^2^ = 0.893, and RMSEP = 0.131). For trilobatin, the model with the best performance was developed with raw NIR spectra (R_P_^2^ = 0.902, and RMSEP = 2.993), and for phloridin, the best model was obtained with NIR spectra preprocessed with standard normal variate (SNV) (R_P_^2^ = 0.818, and RMSEP = 1.085). The coefficients of determination for all calibration sets, validation sets and prediction sets of the best PLS models were higher than 0.967, 0.858 and 0.818, respectively.

**Conclusions:**

The conclusion indicated that NIR spectroscopy has the ability to determine the flavonoid content of sweet tea quickly and conveniently.

## Background

*Lithocarpus litseifolius* (Hance) Chun, which has abundant wild resources in the mountainous region of southern China, is commonly named as “sweet tea” [36]. Sweet tea has been listed as a new food resource by the National Health and Family Planning Commission of China since 2017 [9]. Its leaves, which function as tea, sugar and medicine, have been used as a traditional herbal medicine and tea, and it has the potential to become a functional food and food additive [26]. In sweet tea, the most abundant compounds are polyphenols, with flavonoids are the primary compounds in it [18]. Dihydrochalcones are the main flavonoids in sweet tea entailing trilobatin, phloridin, and other compounds. Flavonoids are the main medicinal ingredients in the sweet tea, which has extensive pharmacological activities, such as anti-diabetes effects, antitumor activity, anticancer activity, antimicrobial activity, anti-inflammatory activity, hepatoprotective activity, antioxidant effects and so on [15, 26, 33].

The principal method to determine the flavonoids content in sweet tea is high-performance liquid chromatography [16]. Although chemical methods can accurately detect the composition and content of substances in sweet tea, their steps are time-consuming, complex and expensive. Thus, these characteristics make the process difficult to popularize and cannot be applied to the rapid detection of flavonoids in sweet tea. Therefore, it is important to establish a cheap, simple and reliable analytical approach for detecting flavonoids content in sweet tea leaves rapidly.

NIR spectroscopy, which uses reflectance in a wavelength range of 700–2500 nm, provides a rapid, convenient, and economical way to determine plant ingredients [29]. NIR spectroscopy is a sensing technology that is largely absorbed by O–H, N–H, R–OH, COOH and C–H bonds, which are the major constituents of organic compounds [32]. The principle of NIR spectroscopy is that the ratio and combination of energy absorbed are different due to the stretching and vibration of different bonds between samples [22]. Therefore, the combinations of peaks and troughs in the NIR spectrum vary from sample to sample because NIR radiation interacts differently with different bonds [23]. Thus, the characteristic information of hydrogen-containing groups in organic molecules can be obtained by scanning the near-infrared spectra of samples [25]. It has been widely used in agriculture, [12, 17, 19], petrochemicals [1, 14], food [28] and pharmaceuticals [6]. The use of NIR spectroscopy in plant leaf tissue analysis started in the mid-1970s [20], and there are an increasing number of papers on NIR spectroscopy application on leaf tissues [4, 13]. Many studies using NIR spectroscopy combined with multivariate calibration methods have analyzed the content of polyphenols and other ingredients in tea [5, 24].

These studies reveal that NIR spectroscopy has the potential to determine compositions in tea. Therefore, in this study, the feasibility of rapidly measuring the flavonoids in sweet tea using NIR spectroscopy was studied. At the same time, to improve the accuracy of the model, different preprocessing methods in combination with PLS were used and compared to determine the most desirable calibration model. NIR spectroscopy is expected to provide a rapid method to measure the flavonoids in sweet tea.

## Methods

### Sweet tea leaf sample preparation

Leaves of sweet tea were collected from different locations, covering major provinces in southern China, including Hunan, Zhejiang, Guangxi, Sichuan, Jiangxi and Fujian. These areas vary in location, soil characteristics, etc. A total of 213 samples were collected to expand the range of variation in the data. The details of different locations of sweet tea leaf samples are outlined in Table 1. Sweet tea leaves were air-dried at the collection sites. Experimental data were derived from three independent repeats for each sample.

**Table 1:** Locations of sweet tea leaf samples

Location	Georeference	Sample number
Lushan, Sichuan	103°52′–103°11′E 30°01′–30°49′N	23
Jinyunshan, Sichuan	106°17′–106°24′E 29°41′–29°52′N	16
Pidu, Sichuan	103°42′–104°02′E 30°43′–30°52′N	4
Pengzhou, Sixhuan	103°10′–103°40′E 30°54′–31°26′N	9
Xupu, Hunan	110°15′–111°01′E 27°19′–28°17′N	58
Jiangshan, Zhejiang	118°22′–118°48′E 28°15′–28°53′N	55
Yiyang, Jiangxi	117°24′–117°61′E 27°99′–28°73′N	5
Wuyuan, Jiangxi	117°21′–118°12′E 29°01′ ~ 29°34′N	14
Bama, Guangxi	107°25′–108°09′E 29°01′–29°34′N	14
Pinghe, Fujian	117°21′–118°12′E 23°53′–24°14′N	15
Total		213

### Chemical measurements

#### Measurement of total flavonoids

The total flavonoid content was measured by a spectrophotometric method [35]. The principle of this method was that under certain conditions, flavonoids react with aluminum to form a red complex, and the color intensity was proportional to the content of flavonoids. Rutin was used as a standard for colorimetry. The absorbance of the standard was measured, and a standard curve was generated. The standard curve was used to calculate the total flavonoid content of sweet tea leaf samples. Briefly, the dried leaves were first ground into powder with a mill. Then, 0.5–1 g of sweet tea leaf powder was weighed and placed into a 250 mL conical flask. A 30 mL extraction solution was used to extract total flavonoids from the powder under supersonic (300 W, Kedao Ultrasonic Instrument Co., Ltd., Shanghai, China) conditions for 1 h. Then, the sample was filtered into a 50 mL volumetric flask while it was hot, and the extraction solution was used to make the volume constant. Next, a 1 mL sample was placed in a 10 mL test tube, 2 mL of 0.1 mol/L ALCL_3_ solution was added, and 3 mL of 1 mol/L potassium acetate solution was added. A 70% methanol solution was fixed to the mark. The solution was shaken well at room temperature for 30 min and used to detect the absorbance value at 420 nm using a spectrophotometer (723 N, Jingke Instrument Co., Ltd., Shanghai, China). Rutin was used as the standard for a calibration curve. The total flavonoid content was calculated using the following equation based on the calibration curve:

A = 28.63C + 0.0023, R2 = 1.

where A is the absorbance and C is the total flavonoid content in mg/mL.

#### Determination of phloridin and trilobatin

Phloridin and trilobatin were determined by high-performance liquid chromatography (HPLC) [16, 31]. First, 0.50 g powder was accurately weighed and mixed with 20 mL 80% methanol solution and ultrasonically extracted for 30 min. After centrifuging at 8000 r/min for 10 min, the supernatant was poured out in a 50 mL volumetric flask, and the process was repeated once. Then, the volume was diluted to 50 mL with 80% methanol solution and passed through a 0.22 μm organic membrane for liquid chromatographic determination. Second, a standard curve was prepared with phloridin and trilobatin standard products and then operated according to the chromatographic conditions to find the peak area and calculate the relevant content according to the standard curve equation. The chromatographic detection conditions were as follows: Waters Atlantis T3 column (5 μm, 250 mm × 4.6 mm); mobile phase: phase A was methanol, phase B was water (V/V = 52/48); the flow rate was 1.0 mL/min; the injection volume was 2 μL; the detection wavelength was 285 nm; and the column temperature was 30 °C.

### NIR spectra collection

The NIR spectra of sweet tea leaves were collected with a handheld fiber optic contact probe from a field-based spectrometer (LF-2500, Spectral evolution, USA). The range of the spectrometer was 1100–2500 nm, and the whole operation was performed at room temperature (approximately 25 °C). Before the leaf sample spectral measurements, a baseline correction was performed with a standard white panel of known absorbance on the absorbance sample port. The powder of sweet tea leaf samples was placed on the sample port closely, and then the absorbance spectrum of each sample was obtained. Three replicates were taken for each sample, and the average was considered. To exclude issues arising from sample mixing, devices were wiped with paper towels after each sample was collected.

### Data analysis

#### Model evaluation

All experimental data was imported into the Unscrambler X software version 10.4 (64-bit) from CAMO (Computer Aided Modeling, Trondheim, Norway). Partial least squares analysis, which is universally applied for calibration in present chemometrics analysis, was performed for the establishment of the flavonoid prediction model. Before the construction of a model, we have scientifically divided a total of 213 samples. The sample set was randomly divided into a calibration set for developing the model and a prediction set to evaluate the built model at a ratio of 2:1 [24]. All the sweet tea leaf samples were sorted from small to large according to chemical content values, and one sample for every three samples is selected as the prediction set samples to test the robustness of the model. In addition, full cross-validation was used to verify the accuracy and stability of the model and the highest number of latent variables was fixed at 15. The main parameters of the PLS models evaluation were as follows: the root mean square error of calibration set (RMSE_C)_, validation set (RMSE_V_), and prediction set (RMSE_P_) and the coefficient of determination of calibration set (R^2^_C_), validation set (R^2^_V_), and prediction set (R^2^_P_). Models with low RMSE and high R^2^ values were considered to be an excellent model [11]. These parameters are presented in the following equation:

1 $$\mathrm{RMSE }= \sqrt{\frac{{\sum }_{i=1}^{n}\overline{y }i-yi}{n}}$$

2 $$\mathrm{R}2\hspace{0.17em}=\hspace{0.17em}1-\frac{{\sum }_{i=1}^{n}{\left({y}_{i}-\overline{y }i\right)}^{2}}{{\sum }_{i=1}^{n}{\left(yi-\overline{y }\right)}^{2}}$$

where, in Eq. (1) and (2), $\overline{yi }$ and yi are the calibration, validation and prediction set values of the samples, respectively, and n is the number of samples.

#### Preprocessing method

The raw spectra not only contain effective information related to target substance content but are also covered by some useless information, that is, the noise and baseline that occurred in the detector and background or other unwanted changes due to the difference in physical structure between samples [34]. Therefore, it is absolutely vital that preprocessing should be done for a good calibration model in spectroscopy analysis. Generally, preprocessing steps on the raw spectra are given below: (1) First, remove noise and baseline; (2) Second, correct the difference between samples. Moving average filters (MA) and Savitzky–Golay (SG) are two methods commonly used for reducing noise. Multiplicative scatter correction (MSC), standard normal variate (SNV) and differential coefficient (first and second derivatives, D1 and D2) are used to correct sample differences. Derivative operations are usually applied in combination with SG. In the present study, to compare different preprocessing methods, six preprocessing methods on PLS were studied, including MA, SG, SNV, MA + MSC, SG + D1 and SG + D2.

## Results

### Variation in the flavonoid content of sweet tea leaf samples among different locations

**Fig. 1:**
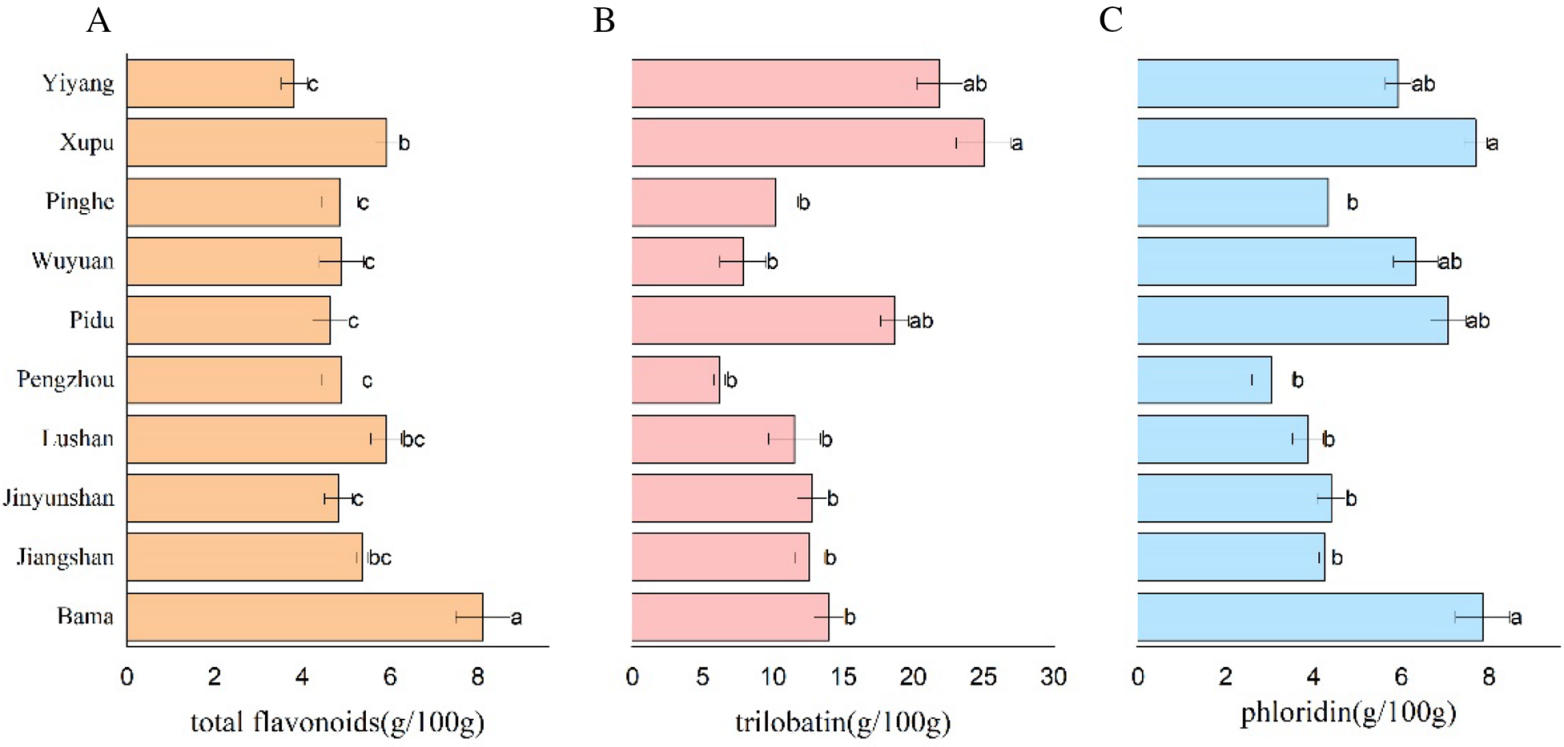
Variation in flavonoid contents of sweet tea samples among different locations

HPLC revealed that the contents of flavonoids in sweet tea leaves from different locations were different. As shown in Fig. 1, sweet tea leaves of Bama, Guangxi, had the maximum levels of total flavonoids and phloridin, 8.12 ± 0.62 and 13.99 ± 1.06 g/100 g, respectively (all values represent means ± sem). The maximum level of trilobation in sweet tea leaves was Xupu, Hunan, with an average of 25.02 ± 1.05 g/100 g.

The correlation coefficients of flavonoid content between different locations are shown in Table 2. It can be used to evaluate the relationship between locations and ingredients. We can see that the content of total flavonoid had a negative correlation with the location (r = − 0.094), phloridin has a significant positive correlation with it (r = 0.159), and trilobatin has a very significant positive correlation with the location (r = 0.272). In addition, trilobatin and total flavonoid showed a very significant positive correlation (r = 0.316) and a very significant negative correlation with phloridin (r = − 0.219).

**Table 2:** Correlation Matrix of constituents between different locations

	Location	Total flavonoids	Phloridin	Trilobatin
Location	1.000			
Total flavonoids	− 0.094	1.000		
Phloridin	0.159^*^	− 0.089	1.000	
Trilobatin	0.272^**^	0.316^**^	− 0.219^**^	1.000

*P < 0.05; **P < 0.01

### NIR Spectra

#### Raw spectra

**Fig. 2:**
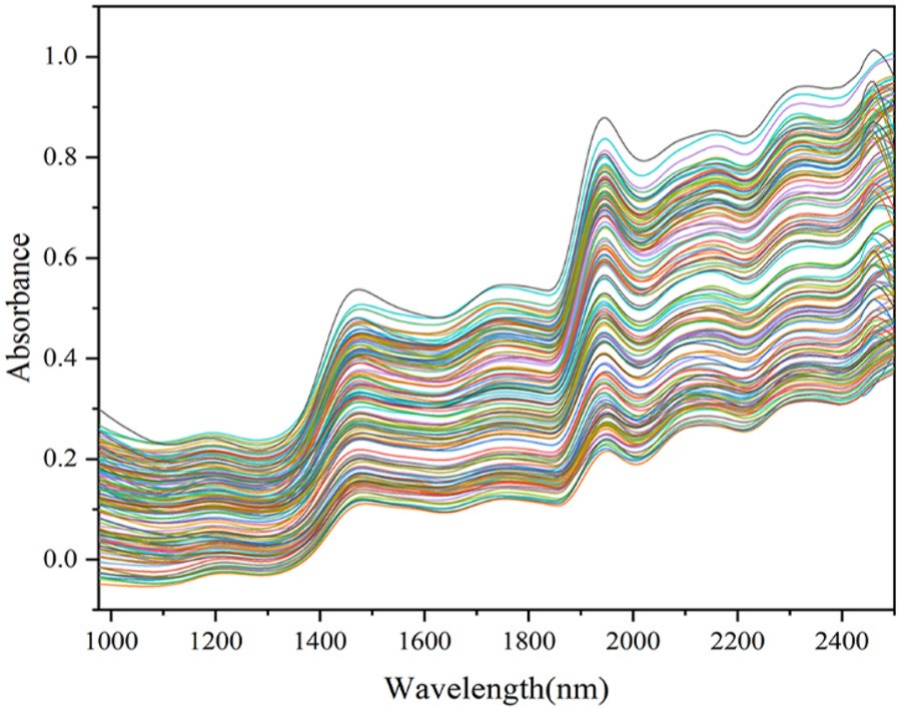
Raw NIR spectra of sweet tea leaf samples

**Fig. 3:**
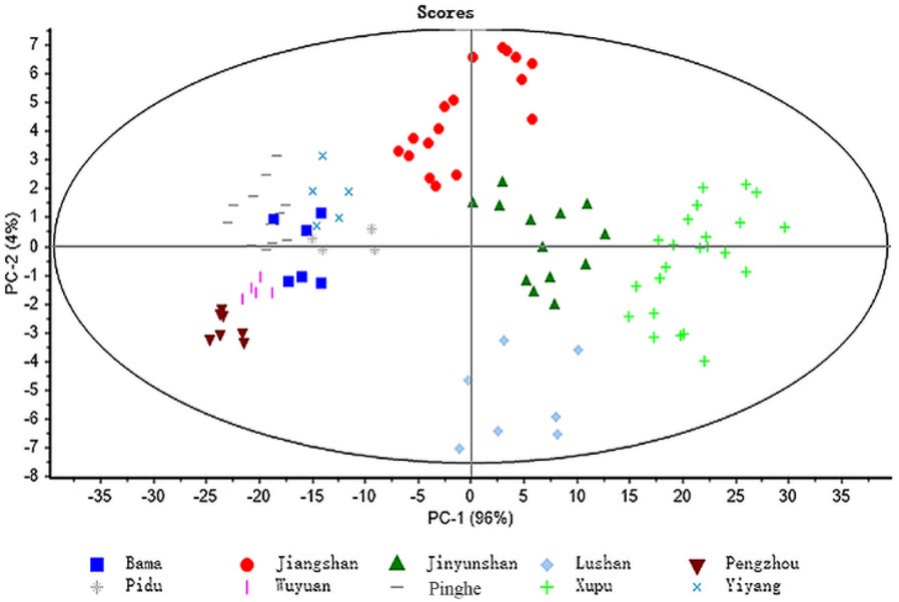
The distribution of all the samples under different locations

The raw NIR spectra of sweet tea leaf samples are shown in Fig. 2. As shown, it was obviously hard to observe the difference between different samples in the original spectra. The spectra had two important peaks. The predominant peaks were related to O–H stretching and bending combinations observed between 1400–1500 nm and 1900–2000 nm [19, 21]. The small plate absorption peaks appearing at 1200 nm, 1650–1800 nm, 2100–2200 nm and 2300 nm were assigned to C-H stretching vibrations [24]. To visualize differences between different locations, principal component analysis was applied to examine the flavonoids of sweet tea from different locations (Fig. 3). After removing the outliers, the result showed that the differences between leaf samples collected in different locations could be reflected by NIR spectroscopy combined with PCA.

#### Spectral preprocessing

**Fig. 4:**
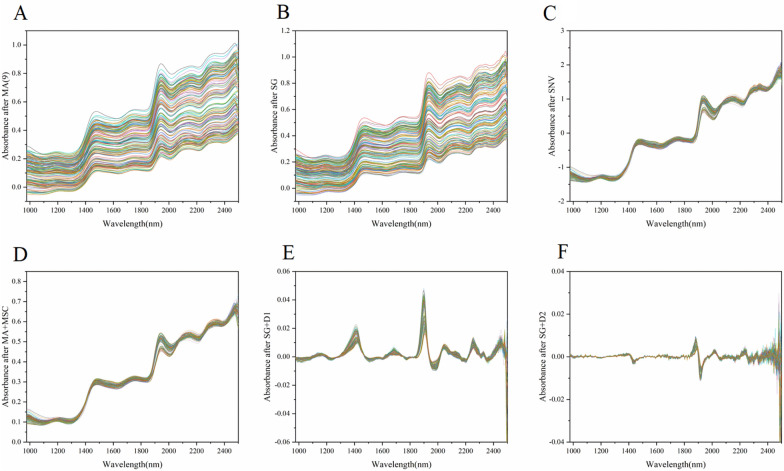
Spectral curves after different preprocessing

Preprocessing methods were applied to the spectra, including MA, SG, MA combined with MSC, SNV, SG combined with first derivative spectra (SG + D1), and SG combined with second derivative spectra (SG + D2). The spectra after preprocessing are shown in Fig. 4.

### Establishment of the PLS model

**Fig. 5:**
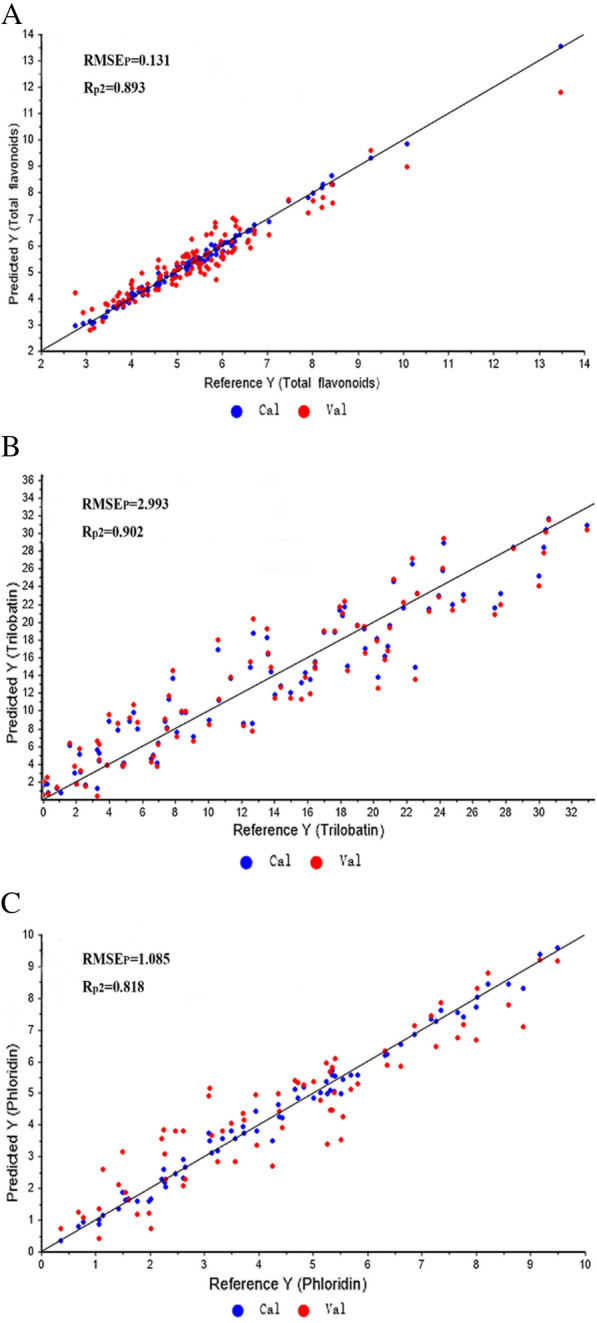
Reference (measured) and predicted values of three constituents in sweet tea leaves

After the calibration set and the prediction set were established, PLS models of the three constituents were constructed with the raw and preprocessed NIR spectra. The evaluated parameters of different preprocessing PLS models are shown in Table 3 to select the model with the best performance. Models with low root mean square error (RMSE) and high coefficient of determination (R^2^) values were considered to be excellent models [11]. Figure 5 showed the prediction results of the PLS models according to which the developed models could predict total flavonoid, trilobatin and phloridin contents.

**Table 3:** The results of the three constituents by PLS models with different preprocessing methods

Parameter						
Method	R^2^c	RMSEC	R^2^v	RMSEV	R^2^p	RMSEP
Total flavonoids						
None	0.826	0.641	0.512	1.085	0.570	0.942
SG	0.751	0.768	0.502	1.095	0.569	0.944
MA(9)	0.708	0.831	0.474	1.126	0.434	1.082
SNV	0.746	0.775	0.408	1.194	0.499	1.017
MA + MSC	0.569	1.010	0.400	1.202	0.412	1.103
SG + D(1)	0.878	0.538	0.453	1.149	0.289	0.522
SG + D(2)	**0.998**	**0.069**	**0.931**	**0.409**	**0.893**	**0.131**
Trilobatin						
None	**0.967**	**1.654**	**0.872**	**3.283**	**0.902**	**2.993**
SG	0.904	3.021	0.831	4.049	0.824	4.412
MA(9)	0.922	2.788	0.851	3.894	0.841	4.059
SNV	0.889	3.274	0.758	4.877	0.792	4.805
MA + MSC	0.837	4.271	0.728	5.564	0.793	4.789
SG + D(1)	0.953	2.083	0.871	3.482	0.194	3.370
SG + D(2)	0.993	0.832	0.829	4.102	0.250	2.457
Phloridin						
None	0.948	0.589	0.752	1.306	0.684	1.378
SG	0.741	1.531	0.482	2.185	0.467	1.906
MA(9)	0.738	1.640	0.531	2.215	0.431	1.891
SNV	**0.987**	**0.263**	**0.858**	**0.893**	**0.818**	**1.085**
MA + MSC	0.776	1.519	0.559	2.151	0.234	2.077
SG + D(1)	0.942	0.686	0.750	1.436	0.139	2.256
SG + D(2)	0.692	1.464	0.432	2.007	0.127	2.368

(Notes: MA, Moving average filters; SG, Savitzky–Golay smoothing with 9 points; MSC, MA combined with multiplicative scatter correction; SNV, Standard normal variate; SG + D1, SG combined with first derivative spectra; SG + D2, SG combined with second derivative spectra; RMSEC, RMSEV and RMSEP, the root mean square error of calibration set,validation set , and prediction set; R^2^_C_,R^2^_V_ and R^2^_P_, the coefficient of determination of calibration set, validation set and prediction set.)﻿

#### PLS model for total flavonoids

As seen from Table 3, the best result of the total flavonoid calibration model was developed with NIR spectra preprocessed with SG + D2. The combination of both, which makes the model more robust and reliable, can play a good role in reducing noise and correcting sample differences [17]. This model, which is shown in Fig. 5A, had the highest Rc^2^ (0.998) and lowest RMSE_C_ (0.069). For the validation set, the best performance was also obtained with SG + D2, with an R_V_^2^ of 0.931 and an RMSE_V_ of 0.409. In addition, the model was tested using the prediction set, and it performed best with a higher R_p_^2^ of 0.893 and a lower RMSE_P_ of 0.131.

#### PLS model for trilobatin

As shown in Table 3, similar to total flavonoids, the best result of the trilobatin calibration model was developed with raw NIR spectra preprocessed with SG + D2. This model had the highest R^2^_C_ (0.993) and lowest values of RMSE_C_ (0.832). For the validation set, the model also had a higher R^2^_V_ of 0.829 and an RMSE_V_ of 4.102. However, a lower performance was observed for the prediction set with lower R^2^ values. The best calibration, validation and prediction PLS models of trilobatin shown in Fig. 5B were developed with raw NIR spectra with R^2^_C_ (0.967), RMSE_C_ (1.654), R^2^_V_ (0.872), RMSE_V_ (3.283), R^2^_P_ of 0.902 and RMSE_P_ of 2.993.

#### PLS model for phloridin

Phloridin content was predicted by the PLS algorithm using different spectral preprocessing methods. According to the calibration set and prediction set of PLS models, the SNV spectral model had the best performance with the highest R^2^_C_ (0.987) and lowest values of RMSE_C_ (0.263). For the validation set, the best performance was also obtained with SNV with an R^2^_V_ of 0.858 and an RMSE_V_ of 0.893. In addition, the model was tested using the prediction set, and it performed best with a higher R^2^_P_ of 0.818 and a lower RMSE_P_ of 1.085. The best performance of the phloridin PLS model is presented in Fig. 5C.

## Discussion

The variation in the flavonoid content of sweet tea leaf samples among different locations was different. The results are consistent with some previous studies [30, 33]. In our research, the content of trilobatin in sweet tea from Hunan was the highest, which is consistent with Yang’s results. In addition, correlation coefficients of flavonoid contents between different locations indicate that there is a certain correlation between the content of the three components in the same leaf. The leaf with higher trilobatin has higher total flavonoids and lower phloridin. In the study of Wei, he found that trilobatin made a large contribution to the tender leaves, and Yang found that the total flavonoid content was highest in the tender leaves, declining as the leaves aged. From previous studies [27, 30, 33], we can conclude that this may be related to the freshness of the leaves, and further research is needed in follow-up experiments.

The results of spectrum preprocessing show that after pretreatment with SG and MA, the spectra did not change much from the original spectrum. The SNV preprocessing method is as good as MA + MSC, which is consistent with the study of Chen [3]. Furthermore, SG + D1 and SG + D2 were considered the best. The results are consistent with Nascimento’s study, which used various preprocessing methods for the spectra, and the first derivative of Savitsky–Golay with five smoothing points was regarded as the best result [19].

Modeling with spectra after different pretreatments, the best PLS model of total flavonoids was developed with NIR spectra preprocessed with SG + D2. Many studies have also reported that the best preprocessing method for NIR spectroscopy is derivatization with SG. For example, Farhadi established a prediction model for starch, reducing sugar, and moisture content in potatoes [8], Zhang measured leaf water content using VIS/NIR spectroscopy [34] and Nascimento developed PLS models for soluble solids content and firmness determination in low-chilling peach [19]. The best PLS model of trilobatin was developed with raw NIR spectra. The results of Hassan’s research also show that non preprocessing was confirmed to be an optimal PLS model to determine flavonoid contents in Chinese wild rice [10].

These results showed that the established PLS models had a good ability to predict the total flavonoid, trilobatin and phloridin contents in sweet tea with high R^2^ and low RMSE values. It shows the correlations between the measured and predicted flavonoids in sweet tea leaves, and the scatter distribution for all constituents is concentrated around the diagonals. NIR spectroscopy has the potential for the rapid prediction of flavonoids in sweet tea. Similar studies for flavonoids have been reported for fresh Ginkgo biloba leaves [7] and propolis [2]. Fang’s model of Ginkgo biloba leaves had a better performance, with an R^2^_c_ of 0.922, an RMSE_c_ of 3.153, a higher R^2^_P_ of 0.905 and a lower RMSE_P_ of 3.511. However, in Betances-Salcedo’s study, the R^2^ values for all prediction sets of the PLS models were between 0.600 and 0.860, and the RMSE_P_ values were between 3.800 and 119.600, which are much lower than those in our research. This may be related to the species, quantity, etc. of the sample.

## Conclusion

The results of this study indicate that a combination of NIR spectroscopy and chemometrics could determine the flavonoid content in sweet tea leaves from different locations. The differences in sweet tea leaf spectra in different locations could be presented by PCA, which created a basis for the quantitative determination of the content of flavonoids by NIR spectroscopy. Furthermore, different spectral preprocessing methods are used in this research to establish the best PLS models between spectral information and the content of the three constituents. NIR spectra preprocessed with SG + D2, none preprocessing and SNV were confirmed to be optimal PLS models to determine total flavonoid, trilobatin and phloridin contents, respectively. The SG + D2 model was achieved with R^2^_P_ = 0.893 and RMSE_P_ = 0.131 for total flavonoid content prediction. For trilobatin content prediction, the none preprocessing model was achieved with R^2^_P_ = 0.902 and RMSE_P_ = 2.993, and the SNV model for phloridin was obtained with R^2^_P_ = 0.818 and RMSE_P_ = 1.085.

NIR spectroscopy is expected to be a rapid, cheaper and convenient method to measure the content of flavonoid in sweet tea. In this study, the flavonoid content determined by NIR spectroscopy was in great agreement with the chemical analysis, indicating that NIR spectroscopy could potentially be used for the quick prediction of flavonoid contained in sweet tea.

## Data Availability

The datasets used and analyzed during the current study are available from the corresponding author on reasonable request.

## Abbreviations

NIR spectroscopy: Near infrared spectroscopy
PCA: Principal component analysis
PLS: Partial least squares
MA: Moving average filters
SG: Savitzky–Golay smoothing with 9 points
MSC: MA combined with multiplicative scatter correction
SNV: Standard normal variate
SG + D1: SG combined with first derivative spectra
SG + D2: SG combined with second derivative spectra
RMSE: Root mean square error
R^2^: Decisive factor
HPLC: High-performance liquid chromatography
